# Successful surgical management of Leriche syndrome in a 30-year-old female patient: A rare case report

**DOI:** 10.1016/j.ijscr.2024.110760

**Published:** 2024-12-27

**Authors:** Lana Sabbagh, Malaka Abubakir, Sana Debajawish, Ghaiyath Khalil, Mohammed Hamdan, Hussein Al-kanj

**Affiliations:** a University of Aleppo, Faculty of Medicine, Aleppo, Syria; b Department of Vascular Surgery, Faculty of Medicine, Aleppo University Hospital, University of Aleppo, Aleppo, Syria

**Keywords:** Leriche syndrome, Aortoiliac occlusive disease, Aortofemoral bypass, Case report, Ankle-brachial index

## Abstract

**Introduction and importance:**

Leriche syndrome (LS), or aortoiliac occlusive disease, is a rare form of peripheral arterial disease leading to claudication, impotence, and diminished femoral pulses due to atheromatous obstruction of the infrarenal aorta and common iliac arteries. Early identification is crucial as untreated LS can result in severe complications. Treatment primarily involves surgical interventions, with endovascular options considered as alternatives.

**Case presentation:**

A 30-year-old former smoker woman presented with calf pain after walking less than 50 m, coupled with a history of recurrent lower extremity arterial occlusions and previous abdominal aorta stenting. Examination revealed pallor and non-palpable pulses in both feet. Doppler imaging and CT scans indicated significant aortic stenosis and iliac artery occlusion. Following a successful bilateral aorto-femoral bypass, distal pulses improved, and the ankle-brachial index was 1 in both limbs. The patient was prescribed warfarin for ongoing management, emphasizing the importance of timely intervention in LS to restore limb perfusion.

**Clinical discussion:**

LS is characterized by atheromatous obstruction of the infrarenal aorta and iliac arteries, causing claudication and absent femoral pulses. Diagnostic tools include the ankle-brachial index, Doppler ultrasound, and CT angiography. Management typically involves aortofemoral bypass or endovascular revascularization, coupled with lifestyle modifications.

**Conclusion:**

Leriche syndrome poses significant morbidity if undiagnosed. This case highlights its impact on quality of life and limb ischemia risk, underscoring the need for accurate diagnostics and timely, personalized management for improved outcomes. Increased clinician awareness is vital for optimal care.

## Highlights

• LS represents a specific type of peripheral arterial disease resulting from atheromatous obstruction of the infrarenal aorta, common iliac arteries, or both.
• Leriche syndrome is characterized by claudication, erectile dysfunction, and diminished or absent femoral pulses.
• LS is classified into three main types
• Diagnostic modalities for LS include the ankle-brachial index, Doppler ultrasonography, and computed tomography angiography.
• Treatment options for LS range from aortofemoral bypass to endovascular revascularization.

## 1 Introduction

Leriche syndrome (LS), also known as Aortoiliac Occlusive Disease (AIOD), is a rare condition characterized by claudication, impotence, and diminished or absent femoral pulses. LS is a specific form of peripheral arterial disease characterized by atheromatous obstruction of the infrarenal aorta, common iliac arteries, or both. It is primarily attributed to the progressive development of atherosclerotic plaques within the aorta [1].

The rates of mortality and morbidity are between 4.5 % and 5.0 % and between 18 % to 20 %, respectively [2].

This condition was first described by Robert Graham in 1814, while French surgeon Reneˊ Leriche was the first to perform surgery for this syndrome, which subsequently bore his name [3]. LS is categorized into three principal types based on the location and extent of atherosclerotic occlusions:

**Type I:** Confined to the distal abdominal aorta and the common iliac arteries.
**Type II:** Primarily involves the distal abdominal aorta with extension into the common iliac and external iliac arteries.
**Type III:** More severe, affecting both the aortoiliac segment and the femoropopliteal arteries [4].

Although LS can be asymptomatic in some cases, it can lead to severe consequences, including mortality, if left undiagnosed or untreated. The risk factors include smoking, hyperlipidemia, diabetes, and hypertension [2].

It is important to consider LS in the differential diagnosis of various vascular conditions, such as arterial dissection in the iliac arteries, which can present symptoms similar to those of Leriche syndrome. Clinical assessment, ultrasound, and CT angiography can be valuable for differentiating the underlying pathology [2]. The primary treatment for LS is surgical intervention; however, angioplasty and endovascular stenting are alternative options for cases with focal involvement [3].

This case discusses Leriche syndrome in a 30-year-old woman. She presented to our hospital complaining of pain in her lower extremities, particularly in the calves, when walking a distance of less than 50 m. The diagnosis in our case was challenging due to the potential misdiagnosis of other conditions, as Lairsch syndrome is relatively uncommon among young females.

This manuscript was prepared by the SCARE 2023 guidelines [5].

## 2 Case presentation

A-30-year-old woman presented to our institute with pain in the lower extremities, concentrated in the calf, which was triggered by walking distance of less than 50 m. Although she had a history of smoking, she had recently quit.

Her medical history was notable for recurrent arterial occlusive in the lower extremities, for which she had undergone stenting due to abdominal aorta occlusion just 8 months prior.

Additionally, her surgical history included five cesarean sections, the last one was 2 years ago, and an appendectomy performed seven years ago. Obstetrically, she had experienced one miscarriage in the second month of pregnancy and the loss of three infants shortly after birth. At the time of presentation, she was taking aspirin 81 mg, rivaroxaban 15 mg, and cilostazol 100 mg.

Upon examination, the patient was conscious, oriented, and responsive, with no signs of bleeding or systemic complaints. However, physical examination revealed pallor in both feet and non-palpable arteries in the lower extremity. While the Abi measured 0.7 in the right limb, and 0.6 in the left limb.

The laboratory results were unmarkable expect the following: Protein C = 50 %, Protein S = 45 %, LA = 47.3 s, ASMA = 56, ana DNA = positive, FOB = (+).

Doppler imaging of the aorta demonstrated reduced color filling, which the iliac arteries displayed a weak waveform resembling a venous pattern in both extremities, indicating obstruction or stenosis within the lumen of the aorta. A computed tomography (CT) scan revealed 30 % stenosis in the abdominal aorta's lumen after the renal bifurcation and confirmed the presence of a 4 cm metallic stent within the aorta. Notably, there was complete occlusion of the aorta's lumen, and there was no visualization of the iliac arteries on both sides. However, collateral circulation had re-established blood flow to the common femoral arteries on both sides. While the remaining arteries of the lower extremities were visualized normally.

**Fig. 1:**
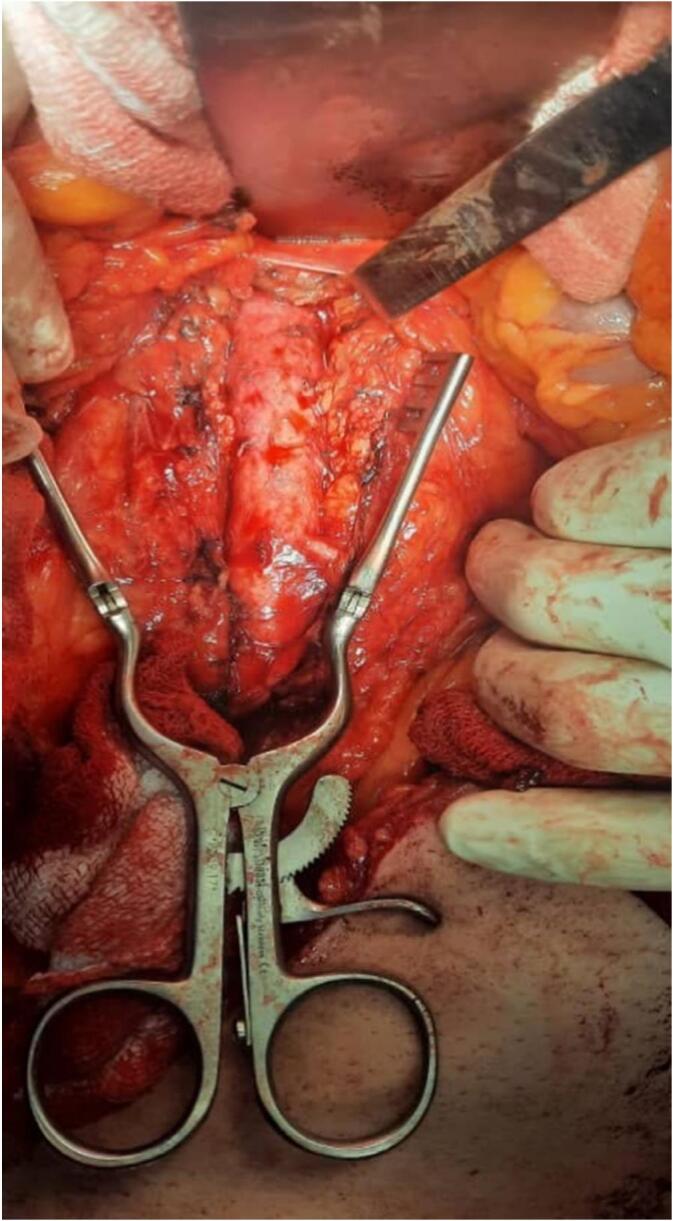
Abdominal aorta isolation.

**Fig. 2:**
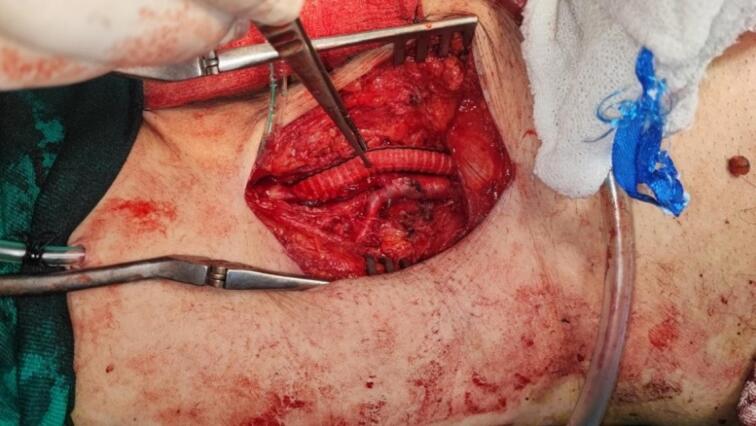
End-to-side anastomosis on the common femoral artery.

The surgical procedure was performed under general anesthesia. The abdominal aorta was isolated below the renal branches (Fig. 1), and the common femoral artery on both the sides was isolated. A bilateral aorto-femoral bypass graft was then placed, accompanied by an end-to-side anastomosis (Fig. 2).

Following the operation, anticoagulation management transitioned to warfarin 5 mg once daily for life due to the patient's persistent thrombotic condition, with adjustment made to maintain an INR between 2 and 3.

Post-operative outcomes included palpable pulse in the ankle arteries of both limbs and the Abi measured 1 in both limbs.

The patient has undergone follow-up for 4 months without any complications or adverse effects appearing.

## 3 Discussion

Leriche syndrome, clinically known as aortoiliac occlusive disease (AIOD), is a significant form of peripheral arterial disease characterized by the narrowing or obstruction of the aorta and iliac arteries in the infrarenal segments. This condition leads to reduced blood flow, which can severely impact the function of the lower limbs [6].

The three primary symptoms of Leriche syndrome (LS) include claudication in one or both lower extremities, diminished or absent femoral pulses, and impotence in males [3]. Additionally, it is important to consider LS when rest pain or ischemic ulcer formation is present [3].

Diagnostic resources for LS include the ankle-brachial index (ABI) examination, which is particularly effective for identifying the presence and severity of AIOD. The ABI is preferred for its non-invasive nature and cost-effectiveness [3,6]. To determine the location and the degree of stenosis, Doppler ultrasonography and computed tomography (CT) angiography must also be used [3,6]. These methods help avoid diagnostic confusion with other arterial diseases that may mimic LS symptoms, such as arterial dissection in the iliac arteries [2].

The primary etiology of LS is atherosclerosis, which is exacerbated by several modifiable risk factors, including tobacco use, hyperlipidemia, hypertension, elevated homocysteine levels, hyperglycemia, and diabetes mellitus. These factors contribute to an increased incidence of the disease. In rarer cases, large vessel vasculitis may also play a role in its development. Non-modifiable risk factors include age, gender, race, and family history [6,7], with a higher prevalence observed in males over the age of 50 [3].

Moreover, LS may be associated with serious comorbidities such as myocardial infarction, heart failure with reduced ejection fraction, dilated cardiomyopathy, and pulmonary embolism [4]. This association underscores the seriousness of LS and its potential to lead to severe complications, including limb ischemia, gangrene, myocardial infarction, and increased mortality [2].

Given these factors, swift diagnosis and rapid intervention are essential. Treatment options vary between aortofemoral bypass and endovascular revascularization [6].

Recent trends favor endovascular interventions for type A and B aortic lesions due to lower costs, fewer complications, and shorter postoperative stays. While device expenses are higher, overall costs are comparable to open surgery, which has longer ICU stays and more adverse events. Open repair shows better patency and survival trends [6,8].

However, aortofemoral bypass remains a valuable option for type C and D lesions [6,9].

Patients scheduled for surgery should cease nicotine use, engage in regular exercise, achieve weight loss, and undergo antiplatelet therapy, along with treatment for hypertension, diabetes, and hyperlipidemia [9].

Patients who are contraindicated for surgery include those who cannot tolerate general anesthesia or who have cardiac or cerebrovascular issues, multiple previous abdominal surgeries, retroperitoneal fibrosis, horseshoe kidney, or end-stage renal disease [9].

The success rate of aortofemoral bypass is approximately 80 %, providing relief from symptoms for up to 10 years [9].

Possible postoperative complications of aortofemoral bypass include cardiac ischemia, renal insufficiency, graft limb thrombosis, anastomotic pseudoaneurysm, and aortoenteric fistula. Additionally, there are common surgical complications such as bleeding, infection, renal or respiratory dysfunction, and myocardial infarction [9].

## 4 Conclusion

In conclusion, Leriche syndrome (LS) represents a serious aortoiliac occlusive disease with the potential for significant complications if not swiftly diagnosed and managed. The case of a 30-year-old former smoker woman with severe claudication underscores the profound impact of LS on quality of life and the risk of critical limb ischemia. Recognizing the multifactorial nature of LS, particularly its association with atherosclerosis, is essential for clinicians. Accurate diagnostic methodologies, such as Ankle-Brachial Index (ABI), Doppler ultrasonography, and CT angiography, are imperative for effective treatment planning. Management should be personalized, with an emphasis on prompt intervention to mitigate morbidity and mortality. Heightened awareness of LS in clinical practice is essential for enhancing patient outcomes and warrants further investigation.

## Abbreviations

LS: Leriche syndrome
CT: Computed tomography
ABI: Ankle-brachial index
AIOD: Aortoiliac occlusive disease

## CRediT authorship contribution statement

HA supervised and helped in writing the manuscript and performed the surgery. LS, MA, GK, SD and MH wrote the manuscript. LS critically revised the manuscript. All authors read and approved the final manuscript.

## Ethical approval

Not required for case reports at our hospital. Single case reports are exempt from ethical approval in our institution.

## Guarantor

Lana Sabbagh.

## Research registration number

Not applicable.

## Consent for publication

Written informed consent was obtained from the patient for publication of this case report and accompanying images. A copy of the written consent is available for review by the Editor-in-Chief of this journal on request.

## Funding

There are no funding sources.

## Declaration of competing interest

There are none to declare.

## Data Availability

All data generated or analyzed during this study are included in this published article.
